# Mechanistic Modeling of a Novel Oncolytic Virus, V937, to Describe Viral Kinetic and Dynamic Processes Following Intratumoral and Intravenous Administration

**DOI:** 10.3389/fphar.2021.705443

**Published:** 2021-07-23

**Authors:** Zinnia P. Parra-Guillen, Tomoko Freshwater, Youfang Cao, Kapil Mayawala, Sara Zalba, Maria J. Garrido, Dinesh de Alwis, Iñaki F. Troconiz

**Affiliations:** ^1^ Department of Pharmaceutical Technology and Chemistry, School of Pharmacy and Nutrition, University of Navarra, Pamplona, Spain; ^2^ IdiSNA, Navarra Institute for Health Research, Pamplona, Spain; ^3^ Merck & Co., Inc., Kenilworth, NJ, United States

**Keywords:** viral dynamics, viral kinetics, mechanistic modeling, oncolytic virus, tumor distribution

## Abstract

V937 is an investigational novel oncolytic non-genetically modified Kuykendall strain of Coxsackievirus A21 which is in clinical development for the treatment of advanced solid tumor malignancies. V937 infects and lyses tumor cells expressing the intercellular adhesion molecule I (ICAM-I) receptor. We integrated *in vitro* and *in vivo* data from six different preclinical studies to build a mechanistic model that allowed a quantitative analysis of the biological processes of V937 viral kinetics and dynamics, viral distribution to tumor, and anti-tumor response elicited by V937 in human xenograft models in immunodeficient mice following intratumoral and intravenous administration. Estimates of viral infection and replication which were calculated from *in vitro* experiments were successfully used to describe the tumor response *in vivo* under various experimental conditions. Despite the predicted high clearance rate of V937 in systemic circulation (t_1/2_ = 4.3 min), high viral replication was observed in immunodeficient mice which resulted in tumor shrinkage with both intratumoral and intravenous administration. The described framework represents a step towards the quantitative characterization of viral distribution, replication, and oncolytic effect of a novel oncolytic virus following intratumoral and intravenous administrations in the absence of an immune response. This model may further be expanded to integrate the role of the immune system on viral and tumor dynamics to support the clinical development of oncolytic viruses.

## Introduction

Over the past 4 decades, the oncology treatment landscape has dramatically changed with the development of advanced and targeted immunotherapies, which complement or even replace classical chemotherapy and radiotherapy strategies (Madden, 2018). Oncolytic virus therapy represents one such novel class of immunotherapy. Advantages of this approach rely on the ability of oncolytic viruses to selectively replicate in cancer cells without harming normal tissues. This process leads to a direct lysis of the tumor mass, as well as the generation of a *de novo* anti-tumor immune response or boosting of an existing one (Kaufman et al., 2015).

At present, talimogene laherparepvec (T-Vec or Imlygic^®^) with intratumoral (i.t) administration is the only FDA-approved oncolytic viral therapy for the treatment of melanoma patients with injectable but non-resectable lesions in the skin and lymph nodes (Pol et al., 2016). Nonetheless, several oncolytic viruses are currently in clinical development to target solid malignancies (Raja et al., 2018). While i. t. administration maximizes the viral load reaching the tumor, it is only feasible for accessible tumors. On the other hand, the intravenous (i.v.) administration would potentially expand oncolytic viral treatment to less accessible tumors as well as obviate the need for interventional radiology, among other benefits. However, systemic administration needs to overcome viral neutralization, liver and spleen sequestration, and vessel extravasation to exceed the a priori unknown “viremic threshold” above which tumor destruction can be achieved (Russell et al., 2012).

To date, there have been only a few published reports describing i. v. administration of oncolytic viruses in clinical trials. In these studies, viral levels as well as biological activities at tumor lesions were observed in some patients (Kaufman and Bommareddy, 2019), however, no evidence for clinical responses have been observed to date (Breitbach et al., 2011; Russell et al., 2014). Furthermore, the currently available data are from early clinical studies with a limited number of patients wherein the endpoints were focused on safety and tolerability rather than therapeutic activity.

In this regard, prior to embarking on large clinical trials, it may be prudent to understand the biology of oncolytic viruses delivery related to tumor response in more detail (Kaufman and Bommareddy, 2019) and mathematical models can provide a quantitative understanding of the biological processes that an oncolytic virus undergoes following different routes of administration.

Various mathematical models to characterize *in vitro* viral dynamics (Bagheri et al., 2011; Titze et al., 2017), *in vivo* behavior limited to tumor size metrics (Bajzer et al., 2008; Eftimie et al., 2011), theoretical frameworks (Wein et al., 2003; Mok et al., 2009; Paiva et al., 2009; Malinzi et al., 2017; Jenner et al., 2018; Cassidy and Humphries, 2019; Al-Tuwairqi et al., 2020) and develop predictive *in-silico* trials (Jenner et al., 2021) have been so far described. To the best of our knowledge, the integration of kinetic parameters including viral distribution to tumor lesions and comparing i. t. and i. v. administrations has not yet been addressed. A greater understanding of these parameters is critical to optimize dosing, schedules, and routes of administration of oncolytic viruses in clinical trial design.

V937 (formerly named CVA21) is a naturally occurring coxsackievirus currently under clinical development. *In vitro* (Shafren et al., 1997a) and *in vivo* (Shafren et al., 2004; Au et al., 2005) oncolytic activity of V937 has been demonstrated in preclinical studies. However, little is known regarding how V937 infection, replication, and tumor distribution *in vivo* relate to the anti-tumor response. The objective of this work was to develop a mechanistic quantitative framework to elucidate the interplay between viral kinetics, dynamics and viral distribution to tumor lesions and then link this information to an anti-tumor response following i. t. and i. v. administration in human xenograft tumor models in immunodeficient mice. The mechanistic model described here will provide the basis to integrate data related to immune response that would allow elucidation of an oncolytic virus driven anti-tumor response under various treatment regimens including combination with immune checkpoint inhibitors.

## Materials and Methods

### Experimental Data

*In vitro* V937 replication data, *in vivo* V937 levels in sera and tumors, and tumor sizes in control and V937-treated mice in multiple human melanoma xenograft studies were integrated in the analysis to build the mechanistic model (raw data depicted in Supplementary Figure 1).

Unpublished in-house data and extracted data from published reports were both processed using R (R Project for Statistical Computing, RRID:SCR_001905) v3.6.1 through RStudio interface (RStudio, RRID:SCR_000432) v1.2.1335. In cases where data were only available from graphs, WebPlotDigitizer (WebPlotDigitizer, RRID:SCR_013996) software was used to extract mean profiles over time. Descriptions of the available data are as follows:

#### *In Vitro* Viral Replication

Data of V937 replication in two human melanoma cells lines SK-Mel-28 (NCI-DTP Cat# SK-MEL-28, RRID:CVCL_0526) and ME4405 (RRID:CVCL_C680) and a rhabdomyosarcoma cell line transfected with ICAM-1, RD-ICAM-1, were extracted from Au et al., 2005. In brief, cell monolayers in 6-well plates were treated with approximately 10^6^ TCID_50_ (half-maximal tissue culture infectious dose) of V937 over 1 h, however the number of seeded cells is not available. At the various time points (0, 2, 4, 6, 8, 10, 12, 24, and 48 h), the cell monolayers were lysed and the viral yield of the cell lysate was determined in triplicate using an end-point titration assay. Average viral titers were digitalized from reported graphs.

#### *In Vivo* Viral Kinetics

Viral RNA measurements of V937 in serum and tumor were obtained from an in-house experiment (Table 1).

**TABLE 1:** Design of *in vivo* experiments.

References	Mice strain	Mice (n)	Cells_0_ (n)	TV_0_ (mm^3^)	Dose	Time points
***Viral kinetic data***						
In-house^a^	SCID	16	2.10^6^	50–150	1) PBS i.v	3, 6, 24, 48, 72, 168, 336, 408 h post-treatment
					2) 10^4^ TCID_50_ i.v	
					3) 10^7^ TCID_50_ i.v	
***Tumor growth data***						
In-house^a^	SCID	4	2.10^6^	50–150	1) PBS i.v	0, 14, 17 days post-treatment
					2) 10^4^ TCID_50_ i.v	
					3) 10^7^ TCID_50_ i.v	
Shafren (Shafren et al., 2004)	NOD SCID	5	2.10^5^	100	1).PBS i.t	0, 8, 14, 21, 30, 35 days post-treatment
	4–6 weeks				2) 10^3^ TCID_50_ i.t	
	NOD SCID 4–6 weeks	5	2.10^5^ per site (2 tumors/mouse)	200–400	1) PBS i.t^b^	1, 17, 23, 30 days post-treatment
					2) 10^3^ TCID_50_ i.t^b^	
					3) PBS i.v	
					4) 10^3^ TCID_50_ i.v	
	NOD SCID 4–6 weeks	5	2.10^5^	400–600	1) PBS i.t	0, 7, 14, 21, 28, 35days post-treatment
					2) 10 TCID_50_ i.t	
					3) 10^b^ TCID_50_ i.t	
					4) 10^3^ TCID_50_ i.t	
					5) 10^5^ TCID_50_ i.t	
Au (Au et al., 2005)	NOD SCID 4–6 weeks	10	10^6^	250–500	1) none	28, 35, 42,49, 56 days post-inoculation

i. v., intravenous (retro-orbital); i. t., intratumoral; n, number of mice or inoculated cells (cells_0_); NOD: nonobese diabetic; PBS: phosphate-buffered saline; s. c., subcutaneous; SCID, severe combined immunodeficiency; TV_0_, tumor volume at the start of the treatment; TCID50: half-maximal tissue culture infectious dose.

a Data from same experiment.

b Dose administered only to primary tumor site.

Ethical review and approval were not required for the animal study as our work presents a modelling exercise on data already published for which the ethical approval was obtained at the time of experiments were performed (by others in other institutions).

Severe combined immunodeficient (SCID) mice were intradermally inoculated with 2 × 10^6^ SK-Mel-28 cells in hind flank on Day −20 for tumor growth. On Day 0, animals were assigned into three groups (n = 16/group) with following treatment regimens: 1) a single i. v. dose of PBS (phosphate-buffered saline), 2) a single retro-orbital dose of 10^4^ TCID_50_ V937 (low dose) 3) a single retro-orbital dose of 10^7^ TCID_50_ V937 (high dose). Sera and tumors were harvested from two animals per group following euthanasia at various time points.

V937 in sera was quantified by both real-time reverse transcriptase polymerase chain reaction (RT-qPCR) (copies/mL) and cell infectivity (TCID_50_/mL), while V937 in tumor samples was quantified by real-time RT-PCR normalized by total RNA (copies/μg RNA). Viral levels were detected in all samples except for two tumor samples harvested at 3 h (low dose V937), and one sample harvested at 17 days (high dose V937).

In this experiment, tumor size was measured using calipers, and the average tumor volumes per group on Day 0 (n = 4), Day 14 (n = 4) and Day 17 (n = 2) were reported.

#### *In Vivo* Tumor Growth Inhibition

In addition to the average tumor volumes obtained from the *in vivo* V937 viral kinetic experiment, tumor size data from two publications (Shafren et al., 2004; Au et al., 2005) were compiled for the analysis. Experimental designs are detailed in Table 1.

In the studies described by Shafren et al., 2004, data were available from three experimental conditions: 1) single i. t. dose of V937 in mice bearing one tumor lesion, 2) single i. t. dose of V937 in one tumor lesion of mice bearing two tumor lesions, and 3) single i. v. dose of V937 in mice bearing two tumor lesions. In all cases, non-obese diabetic (NOD) SCID female mice were subcutaneously (s.c.) injected with 2 × 10^5^ SK-Mel-28 cells in one or two flanks, and single injection of phosphate buffered saline (PBS) or V937 (10–10^5^ TCID_50_) was administered when tumors reached a predefined volume. Tumor volume was calculated using the formula for a spheroid. Tumor volumes, were recorded at regular intervals, and average tumor volume were digitalized for analysis.

*In vivo* growth rate of SK-Mel-28 in NOD-SCID mice following implant of 10^6^ cells in one flank from Au et al., 2005 was combined with control groups in SK-Mel-28 study from Shafren et al., 2004 and used for model building.

In addition to SK-Mel-28 cell line, data from control and treated animals inoculated with ME4405 cells were available from Au et al., 2005. This information was used as validation (see Experimental data section of Supplementary Methods and Results for detailed description of experimental design).

### Mechanistic-Based Model Building

**FIGURE 1:**
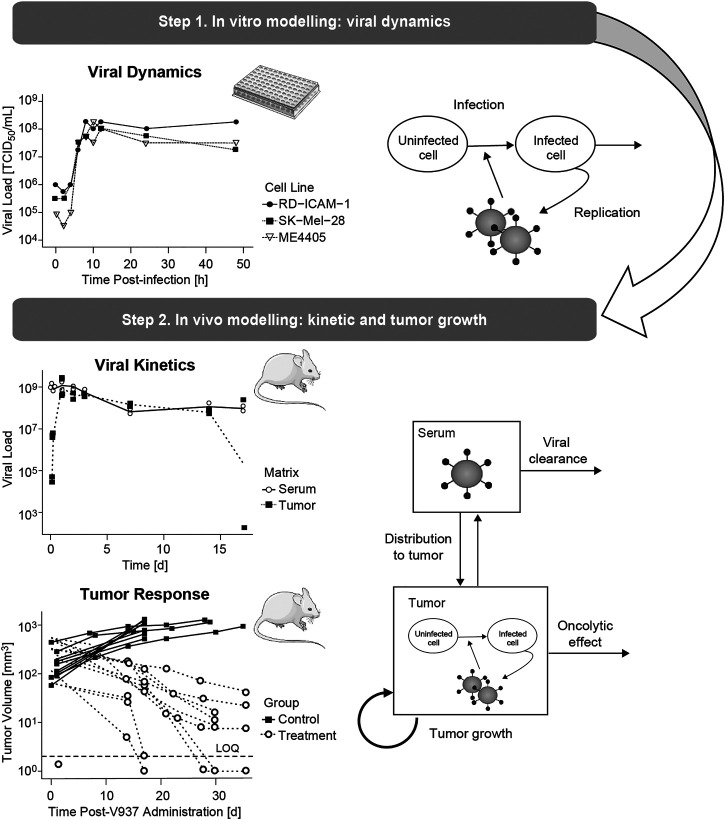
Schematic representation of the modelling and data workflow, highlighting the key processes identified at each step.

The mechanistic model was built following a sequential and integrative approach (Figure 1). First, *in vitro* data were used to characterize dynamics of viral infectivity and replication. Following, *in vivo* viral kinetics, viral distribution in tumor and tumor growth were described taking into account the viral dynamic model previously developed.

**FIGURE 2:**
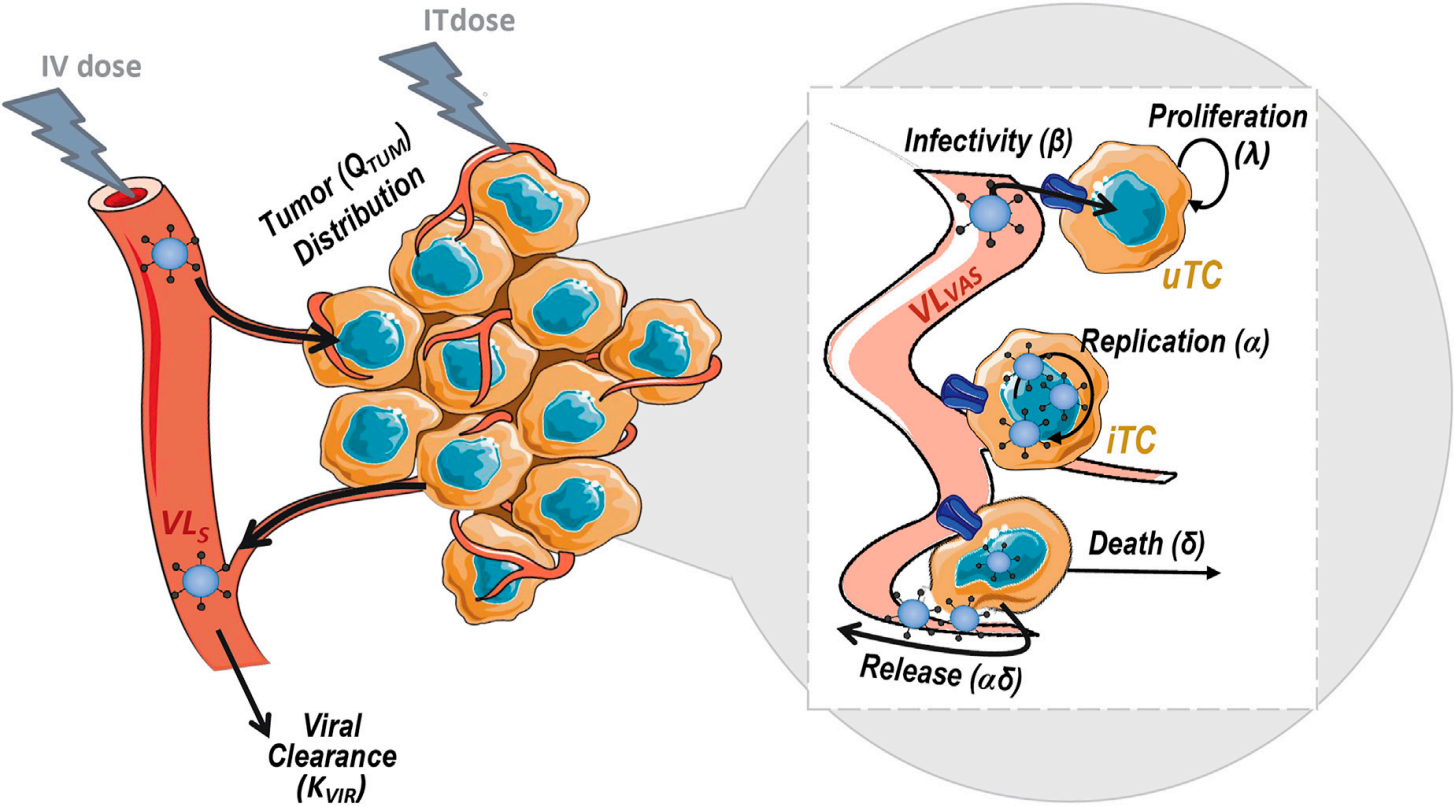
Schematic representation of the mechanistic model for viral kinetics, viral dynamics and tumor growth. uTC, uninfected tumor cells; iTC, infected tumor cells; VL_S_, serum viral load and VL_VAS_, viral load in tumor vasculature.

The model, depicted in Figure 2, accounted for the following biological processes: 1) viral clearance from sera 2) viral distribution to tumor mass, 3) proliferation of tumor cells 4) uninfected tumor cells (uTC) to be infected by the virus, 5) viral replication in infected tumor cells (iTC), and 6) viral induced death of infected tumor cells.

The following subsections describe, 1) the final model structure and parameter estimates, 2) data analyses as methodology used to build the model, 3) model selection and evaluation.

#### Model Structure and Parameters

##### *In Vitro* Viral Dynamic Model

According to the viral mechanism of action and following classical mathematical structures for viral dynamics (Perelson, 2002b), the model described the time course of three main entities: uTC, iTC and viral load in the extracellular medium (VL_EC_) as shown in eqs. 1–3.

uTC can proliferate with a net growth first order rate constant (*λ*) and be transformed to iTC in the presence of virus in the medium. This infectivity process takes place through a second order rate constant (*β*) that consumes both, viruses and uninfected tumor cells. Once cells are infected, V937 can replicate within iTC and generate copies of virions (*α*) per infected cells during its life span. Subsequently, iTC will be killed by viral induced cell death at a rate (*δ*). Extracellular virions can also be degraded with a first order rate constant (K_VIR_).

$$\frac{duTC}{dt}=\lambda \times uTC-\beta \times uTC\times VL_{EC}$$ (1)

$$\frac{diTC}{dt}=\beta \times uTC\times VL_{EC}-\delta \times iTC$$ (2)

$$\frac{dVL_{EC}}{dt}=-\beta \times uTC\times VL_{EC}+\delta \times \alpha \times iTC-K_{VIR}\times VL_{EC}$$ (3)

The system was initialized to the administered dose (TCID_50_) in the compartment of VL_EC_, and number of seeded cells at confluence for uTC (1.2 × 10^6^ cells/well^1^). iTC were considered to be zero at baseline.

Total predicted viral load, which is computed as the sum of viral load in the medium (VL_EC_) and inside cells (*α* × iTS), was fitted to measured viral concentrations from the *in vitro* replication experiment, using the volume of 3 ml per well of a 6-well plate.

Given the short duration of the *in vitro* evaluation, viral degradation and cell proliferation processes were considered negligible, and thus their respective parameters, K_VIR_ and λ, were assumed to be zero at this stage.

##### *In Vivo* Viral Kinetic, Viral Dynamics and Tumor Growth Inhibition Model

To characterize V937 viral kinetics *in vivo*, the time courses of V937 viral load in sera (VL_S_) and tumor vasculature (VL_VAS_) were modeled through a minimum physiological model (eqs. 4–5). Mouse-specific tumor blood flow (Q_TUM_;144 ml/d) (Baxter et al., 1995) and real organ volumes were used in the analysis. Tumor vascular volume (V_VAS_) was assumed to be a 7% of total tumor volume at baseline and serum volume (V_S_ = 0.974 ml) was fixed at 55% (Baxter et al., 1995) of blood volume in mice [77 ml/kg for a 23 g mouse (Mitruka and Rawnsley, 1981)]. Viral elimination rate (K_VIR_) and tumor retention factor representing viral affinity for the tumor (RF) were estimated as drug-specific parameters.

$$\frac{dVL_{S}}{dt}=-Q_{TUM}\times \left(\frac{VL_{s}}{V_{s}}-\frac{VL_{VAS}}{RF\times V_{VAS}}\right)-K_{VIR}\times VL_{S}$$ (4)

$$\frac{dVL_{VAS}}{dt}=Q_{TUM}\times \left(\frac{VL_{s}}{V_{s}}-\frac{VL_{VAS}}{RF\times V_{VAS}}\right)-\beta \times uTC\times VL_{VAS}+\delta \times \alpha \;\times iTC$$ (5)

The initial values of VL_S_ and VL_VAS_ at time 0 were set to the administered dose (TCID_50_) and 0, respectively, following i. v. dose. In the case of i. t. administration, VL_S_ was initialized to 0 and VL_VAS_ to the administered dose at time 0. The kinetic model was linked to the previously developed viral dynamic model at tumor level replacing VL_EC_ by VL_VAS_ in eqs. 1–2 as follows:

$$\frac{duTC}{dt}=\lambda \times uTC-\beta \times uTC\times VL_{VAS}$$ (6)

$$\frac{diTC}{dt}=\beta \times uTC\times VL_{VAS}-\delta \times iTC$$ (7)

As for the *in vitro* model, uTC compartment was initialized to the number of initial tumor cells that were derived from measured tumor size at baseline, and iTC was assumed to be zero at baseline.

To identify K_VIR_ and RF drug parameters, predicted viral load concentration in serum (VL_S_/V_S_ in TCID_50_/mL or copies/mL after correction by a factor accounting for the ratio between TCI50 and copies, referred to as RATIO) and in tumor [computed as the sum of vascular, VL_VAS_, and intracellular levels, *α* × iTS, normalized by an estimated factor to account for the amount of RNA (µg) per tumor volume unit, RNAVOL], were fitted to experimental levels of V937 in sera and tumor over time (see *In Vivo Viral Kinetic Experimental Data* section). The parameters *α* and *β* were fixed to those obtained *in vitro*, while *λ* and *δ* parameters, considered to vary between *in vitro* and *in vivo* experimental scenarios, were estimated by fitting tumor size model predictions (uTC + iTC) to experimental tumor size data (see *Tumor Growth Inhibition Experimental Data* section). Note that tumor size was measured in mm^3^ while uTC and iTC are expressed as cell units, therefore a standard conversion factor of 10^6^ cells per mm^3^ was used (Makkat et al., 2007).

In addition, a proportionality ratio (RATIO) between TCID_50_ and copies was computed to account for the conversion between both metrics as the model describes the dynamics of TCID50/mL and copies/mL, assuming that both variables have same time course but different magnitude.

#### Data Analysis

For *in vitro* or *in vivo* viral levels, each observation was obtained from a different well or animal; therefore, a naïve pool approach was used (i.e. data were pooled together and analyzed as coming from one single ID). For tumor size data *in vivo*, each mean profile was considered as an independent animal, thus enabling the use of the population approach to characterize inter-subject variability in model parameters as well as residual error.

The software NONMEM 7.4 (NONMEM, RRID:SCR_016986) and the First Order Conditional Estimation method with interaction algorithm were used for the analysis.

Data were logarithmically transformed during the analysis and an additive error model in the logarithmic scale was used to account for the discrepancies between model predictions and observations. A different residual error for each measurement was considered. When the population approach was applied the inter-subject variability was modelled exponentially (Kiang et al., 2012; Mould and Upton, 2012).

#### Model Selection and Evaluation

Model selection was largely driven by biological plausibility and capability of the model to describe the tendency of the data, taking into account also 1) parameter precision, 2) classical goodness-of-fit plots including observation versus model prediction and conditional weighted residuals over time or over model predictions, and 3) objective function value (OFV, approximately equal to -2xlog- likelihood). When models were nested, a drop of 3.84 or 6.63 in OFV was considered significant at the levels of 5 and 1%, respectively.

### Model Exploration

The dynamics of the different entities of the system were explored over the range of evaluated dose levels (10–10^7^ TCID_50_), comparing outcome after i. v. or i. t. administration including one or two tumor lesions, the latter allowing for exploration of abscopal effects.

In addition, a parameter scan was performed varying one parameter at a time to explore its impact on viral levels and tumor response for selected design scenarios. During the simulation step,, a function (FMOI) was introduced at the infectivity term: $FMOI\times \beta \times uTC\times VL_{VAS}$; this was used to avoid infectivity of cells at very low virus levels just because high number of uninfected tumor cells in a continuous manner, rather than setting viral levels to zero at random value. This function cancels the infection of new cells when the viruses are too low compared to the number of total cells, with FMOI defined as follows:

$$FMOI=\frac{MOI}{MOI+MOI_{50}}$$ (8)

Where MOI (multiplicity of infection) is computed as the ratio between viral load levels in the vasculature and total tumor burden (iTS + uTS) and MOI_50_ represents the MOI at which 50% of maximum infectivity is obtained. After a sensitivity analysis, MOI_50_ was fixed to a low value (10^–6^) that did not affect model characterization of the experimental data.

Simulations were performed in Berkeley-Madonna software (v9.2.1) and plotted in R (v3.6.1) through RStudio interface (v1.2.1335).

### Sensitivity Analysis

To evaluate the impact of simultaneously varying all parameters on model output, a global sensitivity analysis was performed following the approximation technique described by Saltelli et al., 2008; Saltelli et al., bib_Saltelli_et_al_20102010 of the Sobol’s method (Sobol′, 2001) using the SAFE toolbox available as R package (Pianosi et al., 2015), where to have even probabilities of sampling parameters differing in several order of magnitudes the latin hypercubic sampling method from a uniform distribution was used. Tumor size at day 14 was selected as the variable to represent drug response for model output.

### Model Applicability

The combined impact of the most influential parameters was explored for two-by-two combinations of different dose levels and dosing routes. To do so, a virtual population (n = 1,000) was simulated varying all other parameters but those of interest (i.e. *α* and *β*, *α* and RF or *β* vs RF) following the same sampling approach as described for the global sensitivity analysis.

Tumor size profiles of the virtual population were then simulated using combinations of the two parameters of interest over the plausible parameter space. Probability of response under the different scenarios (i.e. combination of parameters, dose levels and dosing routes) was computed as the probability of observing at least 20% tumor shrinkage at day 14.

## Results

### *In Vitro* Viral Dynamic Model

The levels of viral load obtained *in vitro* showed an initial decay reflecting infectivity, followed by a rapid increase up to 10^8^ TCID_50_/mL, 8–12 h post-infection, indicating viral production (raw data depicted in Supplementary Figure 1A).

Due to the availability of only total viral levels, it was not feasible to account for intracellular viral production and subsequent release to the cytoplasm as independent processes. Describing viral replication as a unique process dependent on both cell death and number of copies per cell allowed us to 1) obtain precise estimates of viral infectivity and production and 2) account for intracellular levels without increasing model complexity. As expected from the quick viral plateau reached *in vitro*, a fast death rate constant (*δ*) was identified, although with low precision due to the lack of cell death time profiles. Accounting for degradation of virus in the medium provided a very low estimate of viral clearance (K_VIR_), suggesting this process could be neglected without affecting model performance (*p* > 0.05), probably as a consequence of the short duration of the experiment.

**FIGURE 3:**
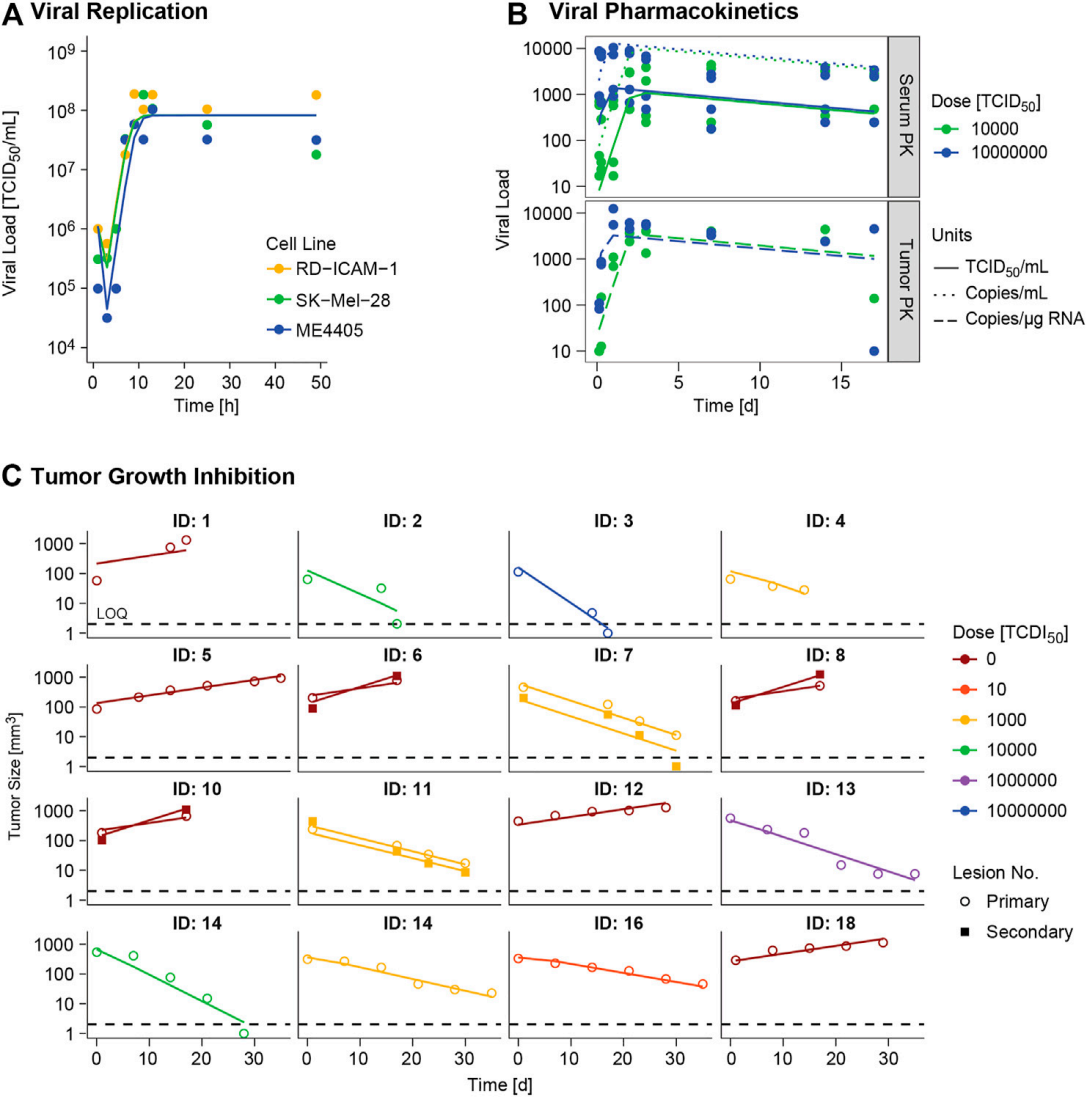
Model evaluation. Model predictions (lines) versus real observations (dots) for **(A)** *in vitro* viral replication model and **(B)** *in vivo* viral kinetic model at typical level, and **(C)** tumor growth inhibition model at individual level for the different experimental scenarios. ID: individual. In panel A viral dynamics of RD-ICAM-1 and SK-Mel-28 are almost equivalent and model predictions appear superimposed.

Different *α*, *β* or *δ* parameter estimates for the three available cell lines (RD-ICAM-1, SK-Mel-28 and ME4405) were evaluated. Best results in terms of model performance and parameter precision were obtained using a different *δ*, estimated to be higher for ME4405 than for RD-ICAM-1 and SK-Mel-28. The use of the different *δ*s provided a significant drop in objective function (*p* < 0.05) and improved parameter precision (from 100% error to less than 60%) (Table 2). Nonetheless, *α* and *β* estimates did not differ statistically across the three cell lines. Overall, a good description of the *in vitro* data was obtained (Figure 3A).

**TABLE 2:** Parameter estimates of final models.

Parameter name	Description	Typical estimate (RSE)	Variability % (RSE)
***Viral dynamics***			
α (TCID_50_/cell)	Viral particles released per infected cell	208 (27.2%)	
*ß* (1/TCID_50_/h)	Infectivity rate	0.489 × 10^−8^ (32%)	
δ_invitro_ (1/h)	Death rate of infected cells *in vitro*	55 (60.7%)	
δ_invitro_ME4405_ (1/h)	Death rate of infected cells *in vitro* for ME4405 cell line	332 (58.4%)	
Error (log TCIDI_50_/mL)	Additive residual error of viral load	0.958 (16.2%)	
***Viral kinetics***			
K_VIR_ (1/d)	Viral degradation rate	232 (14%)	
Q_TUM_ (ml/d)	Blood flow to tumor	144 FIX	
V_S_ (ml)	Serum volume	0.974 FIX	
F_VAS_ (unitless)	Fraction of tumor vascular volume	0.07 FIX	
RF (unitless)	Retention factor of V937 in the tumor	623 (12.4%)	
RATIO (copies/TCI_50_)	Number of copies per TCDI_50_	170 (4.5%)	
RNAVOL (copies x μg RNA/TCID_50_/mL)	Scaling factor to adjust for RNA levels in tumor	1,580 (50.1%)	
Error (log10 TCIDI_50_/mL)	Additive residual error of serum V937 levels in TCID_50_/mL	0.757 (8.8%)	
Error (log10 copies/mL)	Additive residual error of tumor V937 levels	0.807 (8.4%)	
Error (log10 copies/μg RNA)	Additive residual error of tumor V937 viral load	1.29 (11.2%)	
***Tumor growth inhibition***			
λ_1_ (1/d)	Growth rate of primary lesion	0.0601 (6.8%)	
λ_2_ (1/d)	Growth rate of secondary lesion	0.129 (11.1%)	
Tc_0_1_ (mm^3^)	Baseline tumor size of primary lesion	256 (19.1%)	62.5 (20%)
Tc_0_2_ (mm^3^)	Baseline tumor size of secondary lesion	148 (22.6%)	
δ _invivo_ (1/d)	Death rate of infected cells *in vivo*	0.17 (16.1%)	54.6 (21.6%)
Error (log10 mm^3^)	Additive residual error of tumor size in log scale	0.227 (14.1%)	

### *In Vivo* Viral Kinetic and Tumor Growth Inhibition Model

I.V. administration of V937 at both 10^4^ and 10^7^ TCID_50_ resulted in a rapid increase in V937 in sera and tumors, 6–24 h post administration. Despite the 3 log10 difference in dose (10^4^ vs 10^7^ TCID_50_), similar viral concentrations were observed in plateau suggesting viral capability to replicate *in vivo* (raw data depicted in Supplementary Figure 1B).

A semi-physiological pharmacokinetic model that accounts for viral replication in the tumors provided a sufficient description of the available kinetic data in both serum and tumor. Infectivity (*β*) and replication (*α*) estimates were fixed to those obtained from the *in vitro* analysis, while physiological parameters (i.e. tumor blood flow, serum volume and fraction of tumor vasculature) were taken from the published literature. The rest of the model parameters were estimated. High V937 elimination (t_1/2_KIV_ ∼4.3 min) and V937 tumor retention (RF), supported by the low initial viral levels and the capability of the virus to efficiently distribute to tumor and quickly replicate, were identified with good precision.

Regarding tumor size data, certain dose response could be identified, but no major differences across routes were experimentally detected, though large variability in tumor size at dosing time was observed (raw data depicted in Supplementary Figure 1C). When simultaneously modelling control and treated groups, exponential growth provided a better overall description (*p* < 0.001). Variability across mean tumor profiles was identified on baseline tumor cells and death rate of infected tumor cells. In addition, estimating different baseline and growth rate for secondary tumor lesions produced a significant (*p* < 0.01) decrease in OFV.

Acknowledging that tumor growth/shrinkage could also impact V937 viral distribution to the tumor (i.e. tumor blood flow), different models that assumed changes in Q_TUM_ with tumor mass were explored either assuming a proportional relationship between them (i.e. tumor blood flow and tumor mass) or as a power model that allow to account for the fact that not all tumor mass is perfusable (Ferl et al., 2005). None of the models was supported by the data, neither provided a different description of pharmacokinetic (PK) profiles and therefore this feature in the model was not included.

Overall, the final model was able to satisfactorily describe all sources of *in vivo* data simultaneously (Figures 3A–C) enabling precise parameter estimates (Table 2). Moreover, the final model structure could adequately describe the limited experimental tumor response data obtained when implanting ME4405 cell line in xenografts (see results section of supplementary material).

### Model Exploration

The final model was used to explore the behavior of the observed and unobserved elements of the system under different experimental scenarios.

**FIGURE 4:**
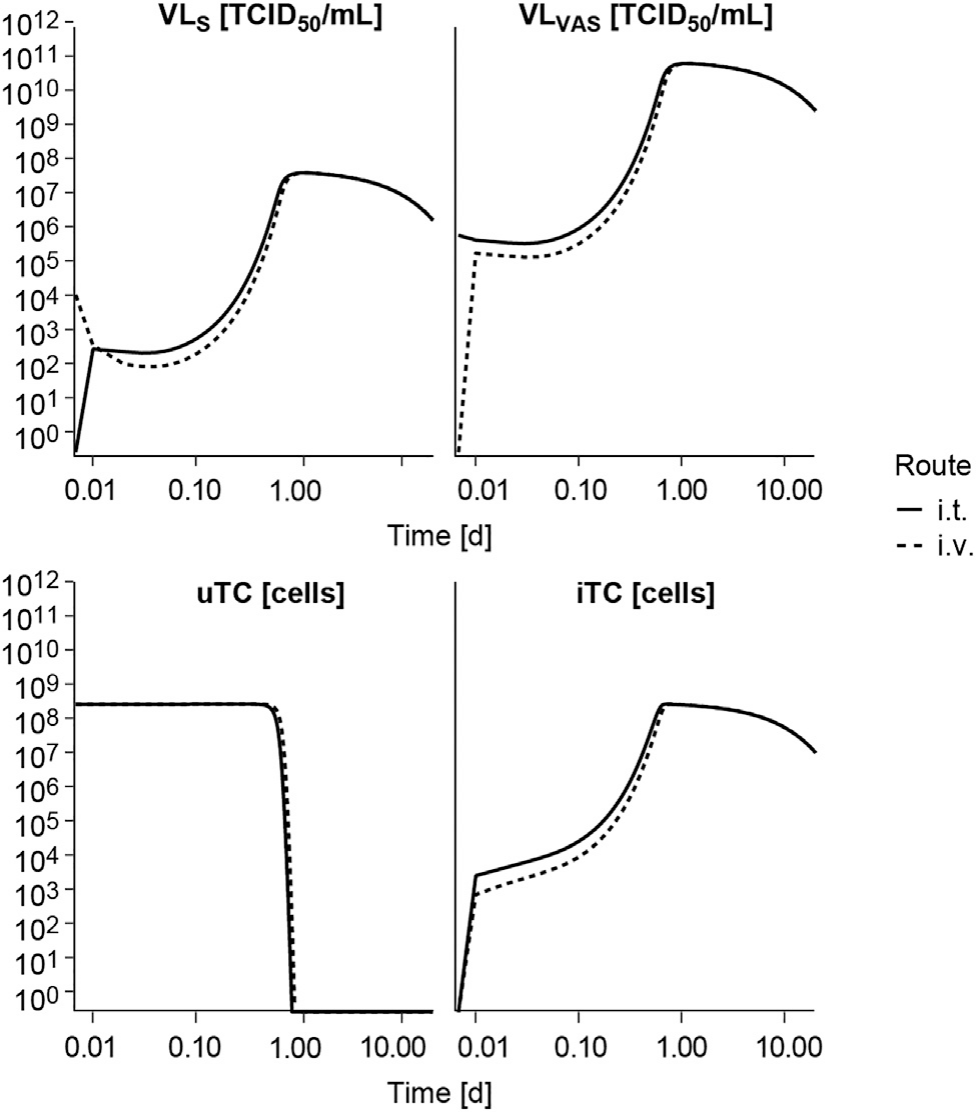
Model exploration. Model predicted time course of the different entities following intravenous (i.v.) or intratumoral (i.t.) single administration of V937 at a dose levels of 10^4^ TCID_50_. VL_S_, viral load in serum; VL_VAS_, viral load in tumor vasculature; uTC, uninfected tumor cells; iTC, infected tumor cells. Log-log scale used.

Following i. v. or i. t. administration, V937 reaches a quick equilibrium of distribution between serum (VL_S_) and tumor vasculature (VL_VAS_) as shown in Figure 4 and at both at the primary and secondary lesion as shown in Supplementary Figure 2. Given the estimated high tumor affinity and infectivity, the model predicts that the vast majority of cells get quickly infected (in less than 1 h at a dose of 10^4^ TCID_50_) and start to produce new viral copies that return to serum. This aspect, observed at all experimental doses available (Supplementary Figure 3), explains the increment in observed circulating levels, despite the fast viral degradation. Time to reach the maximum infectivity of tumor cells, and thus peak viral load levels, depends on both dose and initial tumor burden, with a shortening in time observed as dose level (Supplementary Figure 3) or tumor size increases (data not shown). Under these conditions, differences between routes of administration are mainly located at early time points, before maximum infectivity is achieved, but in all cases tumor response is ultimately attained, as also observed experimentally.

### Sensitivity Analysis

**FIGURE 5:**
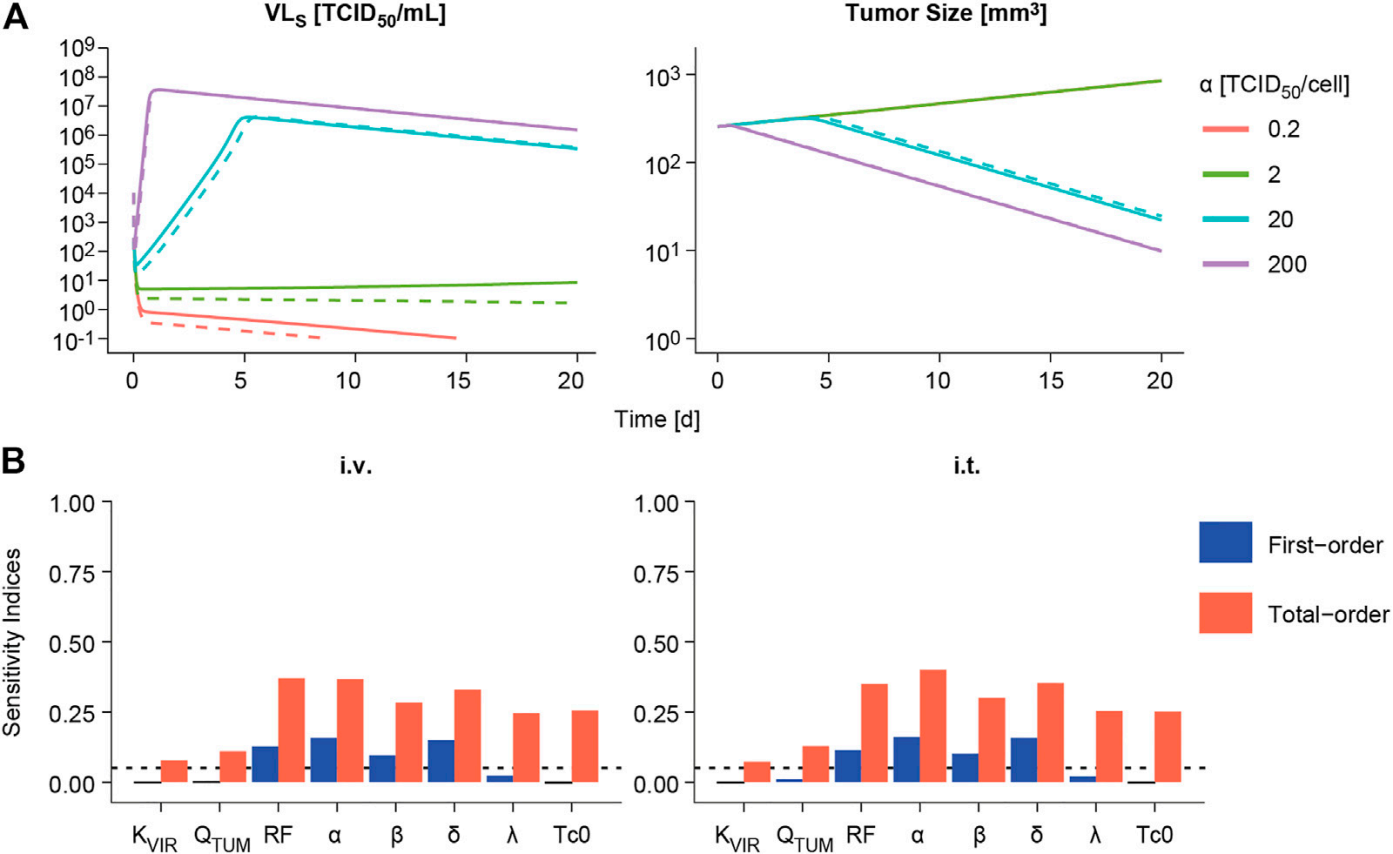
Sensitivity analysis. **(A)** Impact of varying viral replication (*α*) parameter on the predicted time course of viral concentrations in viral load in serum **(**VL_S_, **left panels)** and tumor size volume **(right panel)** following intravenous (dashed line) or intratumoral (solid line) administration of a single V937 dose of 10^4^ TCID_50_. **(B)**. First-order and total-order Sobol’s sensitivity indices computed using model predicted tumor size at day 14 following intravenous (i.v.) or intratumoral (i.t.) single administration of a V937 dose of 10^4^ TCID_50_.

The impact of varying one model parameter at a time within a plausible parameter space was evaluated as exemplified for α in Figure 5A and for the rest of model parameters in Supplementary Figure 4. Only changes in viral replication (*α*), viral infectivity (*β*) or tumor retention factor (RF) were able to invert the course of tumor response from cure to progression when parameter values were decreased. The rest of parameters showed an impact on the rate of response, like the infected cell death rate (*δ*), or minor influence on overall profiles, without changing tumor progression.

When performing a global sensitivity analysis to simultaneously explore the impact of model parameter changes on tumor response, similar results were obtained (Figure 5B). *α*, *β*, RF and *δ* were the most influential parameters, accounting for around 10–16% of the variability in response as single parameters (first-order index), and 28–40% in total when taking into account the different doses evaluated and routes of administration (Supplementary Figure 4). These data highlight the importance of combined effects among parameters to explain model outcome. However, a different scenario is observed for the lowest dose of 10 TCID_50_; this is due to the lack of infectivity and response for most of the simulated parameter vectors, thus leaving the rate of tumor growth and baseline tumor size as the most influential parameters determining tumor size outcome.

### Model Applicability

The model was further evaluated to study the probability of observing a response (i.e. tumor shrinkage greater than 20%) for different two-by-two combinations of most influential model parameters with an impact on ultimate response.

**FIGURE 6:**
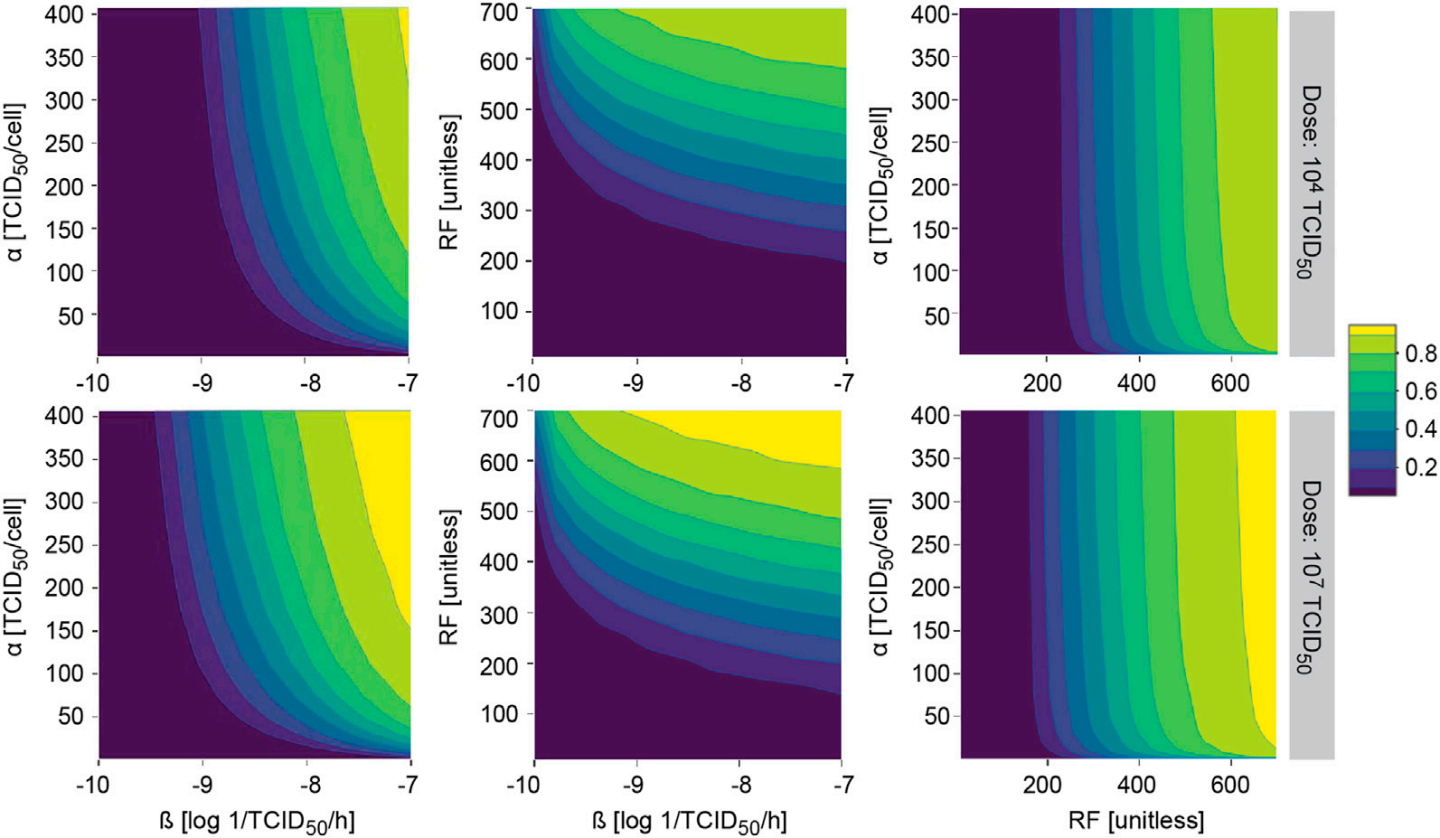
Model applicability. Probability of observing at least 20% of tumor shrinkage at day 14 in a simulated virtual population at different two-by-two parameter combinations after single intratumoral dose. *α*, viral particles released per infected cell (viral production); *β*, viral infectivity; RF, retention factor.

This methodology allowed us to identify those areas where the probability of observing a response is almost null or very high, regardless of the combination of all other model parameters. As expected under this system, similar profiles were obtained after i. v. and i. t. administration, with slightly higher probability of response for the latter (data not shown). In all explored scenarios, increasing the dose translated into an increase in the probability of response, covering a wider area of the parameter space (Figure 6).

## Discussion

Mathematical modelling in immunology and virology has been extensively explored to provide a better understanding of the infection time course and to optimize viral therapies (Perelson, 2002a; Santiago et al., 2017). With the increasing interest in the use of oncolytic viruses in treating cancer, different models that integrate these concepts into a tumor dynamic environment have appeared (Santiago et al., 2017).

In this work, we present a modelling framework using ordinary differential equations that is capable of simultaneously accounting for three key processes of a novel oncolytic virus, V937, currently in clinical development: 1) capability to infect and replicate within tumor cells, 2) pharmacokinetics including distribution of V937 virus to tumor and 3) finally oncolytic V937 effects *in vivo*. To the best of our knowledge, this is the first time that distribution to tumor has been explicitly taken into account to enable characterization of different routes of administration.

To build the framework, an intermediate approach between the classical top-down and bottom-up approaches (i.e. data driven vs biology driven) was followed, thus balancing mechanistic knowledge and available data. Data from different sources, levels of information and even measurement units were integrated, highlighting the importance of quantitative models to combine and exploit experimental data as already shown in other therapeutic scenarios (Campagne et al., 2018; Parra-Guillen et al., 2020).

In this respect, the use of *in vitro* data to identify viral infectivity and replication processes, which are hard to identify only using *in vivo* data, has been of particular relevance. Parameter comparison with other viruses is not straight-forward as different dosing and measurement units (pfu, copies, TCID_50_) are used across experiments. However, in absolute terms, V937 replication capability was within the reported ranges for oncolytic viruses of 50–1,350 virions/cell (Liu et al., 2000), which in most cases come from direct experimental observations and not from modelling exercises. Similarly, estimated *in vitro* death rate was in line with the upper ranges reported by Titze et al., 2017 (1.2–761 1/h) using a similar model structure, as well as the infectivity rate (0.35 × 10^−8^ to 0.98 × 10^−8^). Nonetheless, larger intervals depending on the virus and cell type can be found in the literature for viral infectivity (10^−7^–10^–10^) (Bajzer et al., 2008; Mahasa et al., 2017; Cao et al., 2018).

V937 employs the intercellular adhesion molecule I (ICAM-I, CD54) receptor for attachment and viral entry (Shafren et al., 1997a; 1997b); this receptor is overexpressed in numerous malignant cells, including melanoma (Kageshita et al., 1993; Hayes and Seigel, 2009). V937 can also bind to the DAF receptor; however, it appears that DAF may function as a low-affinity attachment receptor either enhancing viral presentation or providing a viral sequestration site for subsequent high-affinity binding to ICAM-1 (Shafren et al., 1997b). Despite different ICAM-I expression in the three *in vitro* cell lines, differences in viral entry across cell lines could not be adequately identified. This could potentially be due to the experimental data and design, e.g. the lack of dose range as well as errors associated with end point titration for TCID_50_ assessment. However, it could also be due to a sufficiently high ICAM-1 expression levels in all cases (Au et al., 2005) compared to the threshold of 5,000 ICAM-1 molecules per cell required for V937 to exhibit *in vitro* activity (Annels et al., 2018). Nonetheless, and given the mechanistic nature of the model, additional data for example regarding ICAM-I expression could be easily introduced to further refine the model.

In the current model tumor cells infection is predicted to take place quickly and in less than a day due to either the high infectivity rate constant obtained *in vitro* in combination with the high predicted tumor levels after i. t. administration, or the fast tumor distribution and high tumor retention estimated after i. v. distribution. That critical aspect of the model has been explored carefully and is supported by the data. Specifically, viral replication *in vitro* indicated a fast infectivity process, with increasing viral levels reaching a plateau 8–12 h after V937 exposure. Moreover, it has been described that the virus is capable to trigger an extensive lytic destruction of melanoma cells 23 h after infection at a dose level of one TCID50/cell (Shafren et al., 2004), level similar to the one used *in vitro*. Regarding the vivo scenario, a sharp and sustained increase in viral levels at tumor is also observed after i. v. administration, despite the high viral clearance, supporting that infection and replication of the virus takes place fast, given the current model structure.

One novel aspect introduced in this work is the description of viral kinetics and viral distribution to tumor using a semi-physiological pharmacokinetic model that enables simultaneous description of both i. v. and i. t. administration routes. Viral measurements at tumor level are scarce in the literature (Kelly et al., 2008; Workenhe et al., 2014; Garcia-Carbonero et al., 2017; Andtbacka et al., 2019), and even more so over time. However, these types of data have been essential to characterize the tumor compartment in the model. In our modelling framework, the tumor was represented by two sub-compartments: 1) a vascular compartment in fast equilibrium with serum and 2) a cellular compartment which gets infected upon viruses arriving to the vascular compartment. A high retention of the virus at tumor level (RF = 623), which cannot be compared due to lack of literature data, and direct release of newly formed virions to the vascular compartment were required to successfully characterize the data behavior, suggesting no distribution limitations between serum and tumor in our preclinical setting. Moreover, an estimated half-life of 4.3 min, in line with the fast clearance observed for oncolytic viruses in clinical studies (Garcia-Carbonero et al., 2017), was obtained. This result indicates that the sustained levels observed in tumor and plasma are due to V937 capability to infect and replicate in immunodeficient mice, which ultimately leads to a potent oncolytic response as reflected by the infected cell death rate constant (*δ*) estimate, which is in line with those reported in the literature (Okamoto et al., 2014; Cao et al., 2018).

One of the major hurdles in current drug development in oncolytic viruses, and immune-therapies in general, is the need for predictive animal models (Russell et al., 2012). Certainly, xenograft mouse models that lack an immune system can be limited. However, they present a valuable opportunity to assess the properties of the oncolytic viruses in isolation, without any other limitation, on human tumor cell lines, as illustrated in this work. In this mouse model, viral retention, infectivity and replication at tumor level were identified as the key processes controlling V937 tumor response. These processes are tightly interconnected and difficult to identify simply using tumor response data. Viral infectivity and replication identified *in vitro* experiments could be directly integrated into the *in vivo* model structure to enable an adequate prediction of tumor response, thus providing an *in vitro*/*in vivo* framework for oncolytic viruses that can be used to support the selection between candidates based on their *in vitro* properties.

In this tumor mouse model and despite the rapid systemic clearance, minor differences between i. v. and i. t. routes of administrations were observed due likely to high viral infectivity and replication in the tumor level, as reflected by the corresponding parameter estimates, which in principle can lead to complete tumor eradication. However, this result should be interpreted with caution at the time to translate to other scenarios as the impact of the immune system is not yet considered and its role on the viral infection and proliferation mechanisms cannot be ruled out. In addition, given the small number of animals used in the experiments further data would be needed to validate the current model structure and parameter estimates. In this regard, model development should be seen as an iterative process that needs to be coupled with experimental work in order to reflect biology of the system and maximize model usefulness. As a next step, information from syngeneic mouse models that include not only tumor size measurements, but also kinetic levels in serum (and ideally in tumor as well) and relevant immune response markers, could be integrated into this framework. Such developments could facilitate a mechanistic and quantitative understanding of the dual role that the immune system can play in viral response, potentially limiting viral infectivity as well as triggering a potent anti-tumor immune response.

In summary, a mechanistic framework integrating *in vitro* viral dynamic properties into an *in vivo* system to describe oncolytic effects of V937 on tumor response in immunodeficient mice has been successfully developed. This model allows for a better understanding of the role that the different processes play in the final outcome, and enables selection between oncolytic virus candidates based on *in vitro*/*in vivo* features, such as infectivity. Moreover, the developed model can serve as a backbone to include future additional biological components, such as the immune response, to provide a quantitative understanding of the balance between immune and antiviral response. This would facilitate a better understanding of the limitations of systemic administration in immunocompetent scenarios, guide dosing strategies, and help identify potential combination strategies to ultimately support the development of programs for oncolytic viruses.

## Data Availability

The original contributions presented in the study are included in the article/Supplementary Material, further inquiries can be directed to the corresponding author.

## Footnotes

1
https://www.thermofisher.com/es/es/home/references/gibco-cell-culture-basics/cell-culture-protocols/cell-culture-useful-numbers.html
