# Supplementary material for: Mechanistic Modeling of a Novel Oncolytic Virus, V937, to Describe Viral Kinetic and Dynamic Processes Following Intratumoral and Intravenous Administration
Source: Front Pharmacol. 2021 Jul 23;12:705443. doi: 10.3389/fphar.2021.705443 (PMC8343024; doi:10.3389/fphar.2021.705443)
Supplement: Supplementary file 1 [file DataSheet2.PDF]

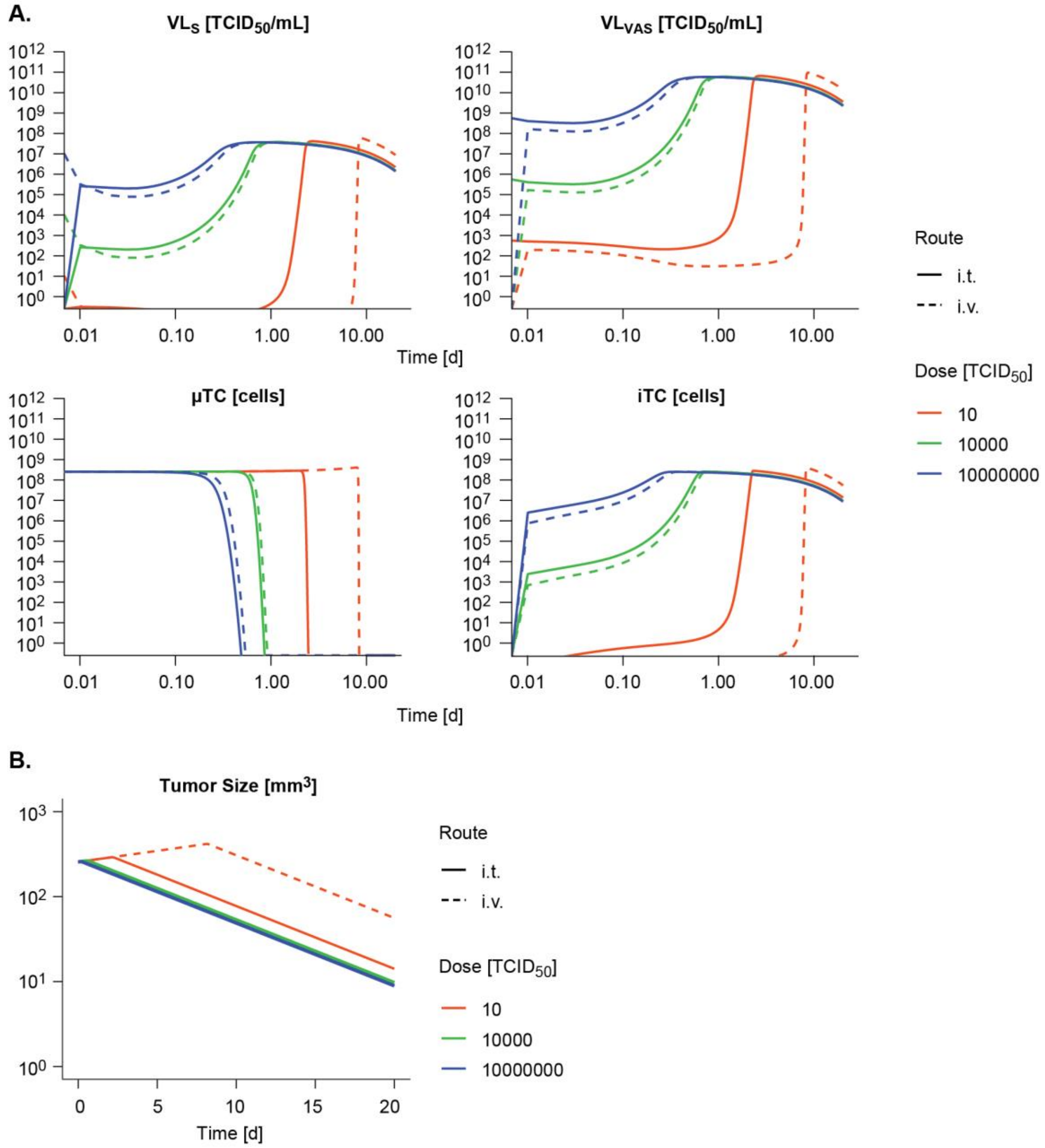

Supplementary Figure 3: Model exploration. Predicted time course of (A) the different model entities or (B) the model predicted tumor size (mm^3) after single intravenous (dashed line) or intratumoral (solid line) administration over a wide range of doses [TCID$_{50}$] of model entities VL$_S$: viral load in serum, VL$_{VAS}$: viral load in tumor vasculature, VL$_{INTRACELLULARS}$: viral load in tumor cells, uTC: uninfected tumor cells, iTC: infected tumor cells. Log- log scale used in panel A only.
