# Supplementary material for: Mechanistic Modeling of a Novel Oncolytic Virus, V937, to Describe Viral Kinetic and Dynamic Processes Following Intratumoral and Intravenous Administration
Source: Front Pharmacol. 2021 Jul 23;12:705443. doi: 10.3389/fphar.2021.705443 (PMC8343024; doi:10.3389/fphar.2021.705443)
Supplement: Supplementary file 2 [file DataSheet4.PDF]

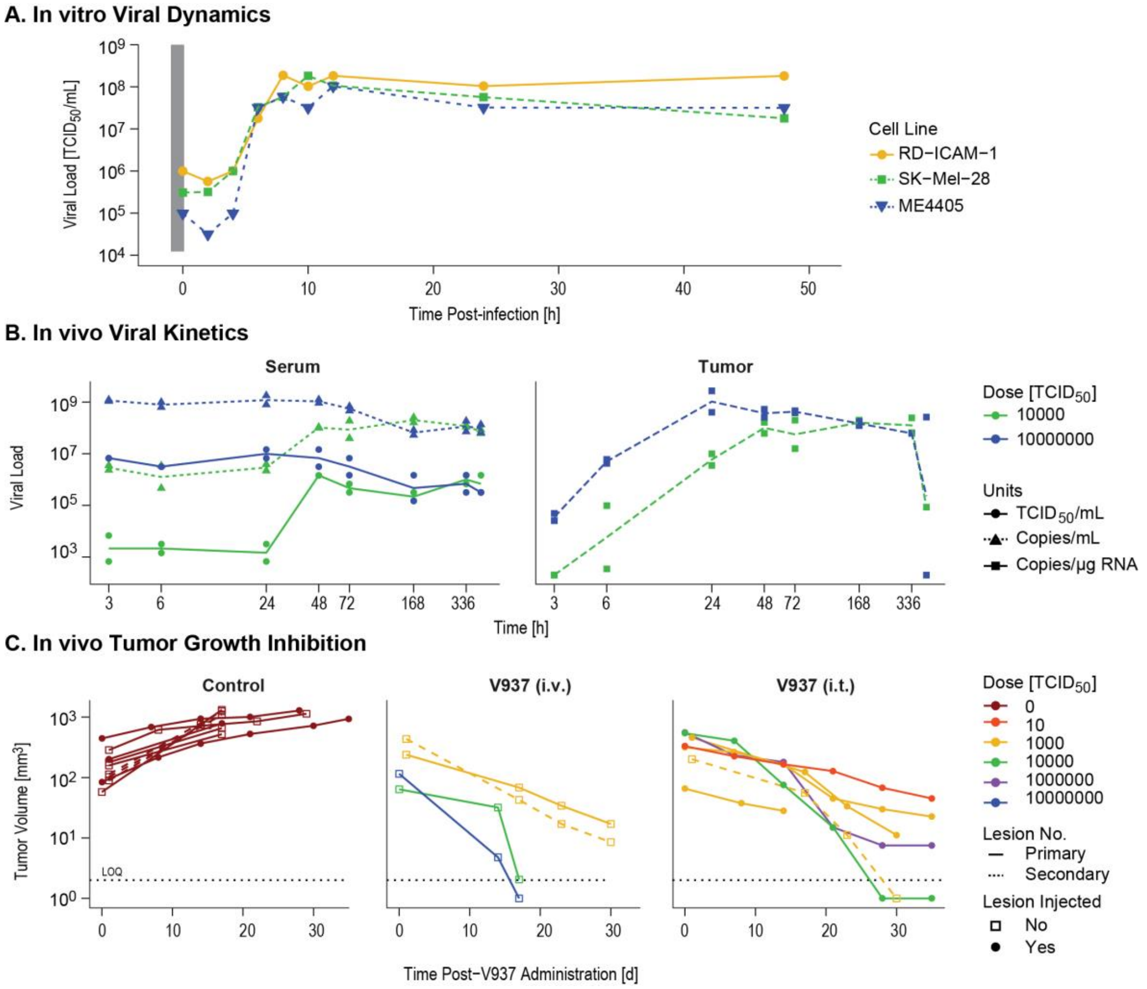

Supplementary Figure 1: Raw Data. A) In vitro viral replication dynamics after cell exposure to a V937 dose of 106 over 1h (grey vertical rectangle). B. Viral load time course in serum and tumor after single intravenous administration to SK-Mel-28 tumor bearing mice (n=16, from in-house studies). C) Tumor volume time course after PBS administration control (n=7; in-house (n=2), Shafren et al. (n=5)) or single intravenous (i.v.; n=3; in-house (n=2; values correspond to an average of 4 or 2 measurements (days 0, and 14, and 17, respectively), or Shafren et al. (n=1)) or intratumoral (i.t.; n=6 from Shafren et al.) administration to SK-Mel-28 tumor mice bearing 1 or 2 tumor lesions (intratumoral administration given to primary lesion only) over a wide range of viral doses. Tumor volume profiles up to 17 days correspond to in house data while longer ones were obtained from Shafren et al.
