# Supplementary material for: Mechanistic Modeling of a Novel Oncolytic Virus, V937, to Describe Viral Kinetic and Dynamic Processes Following Intratumoral and Intravenous Administration
Source: Front Pharmacol. 2021 Jul 23;12:705443. doi: 10.3389/fphar.2021.705443 (PMC8343024; doi:10.3389/fphar.2021.705443)
Supplement: Supplementary file 3 [file DataSheet3.PDF]

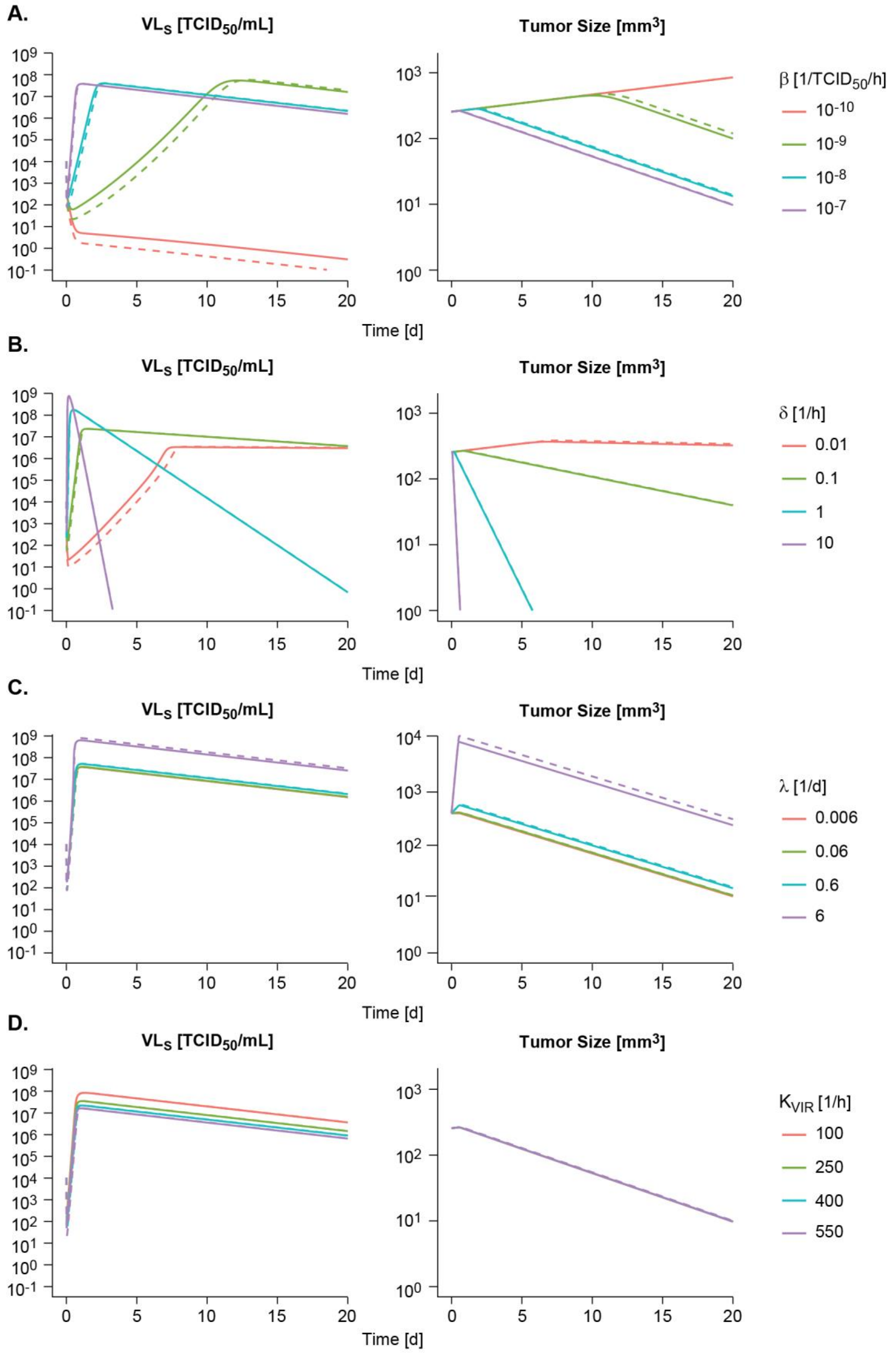

A.
VLS [TCID50/mL]
Tumor Size [mm3]
β [1/TCID50/h]
10-10
10-9
10-8
10-7
Time [d]
B.
VLS [TCID50/mL]
Tumor Size [mm3]
δ [1/h]
0.01
0.1
1
10
Time [d]
C.
VLS [TCID50/mL]
Tumor Size [mm3]
λ [1/d]
0.006
0.06
0.6
6
Time [d]
D.
VLS [TCID50/mL]
Tumor Size [mm3]
KVIR [1/h]
100
250
400
550
Time [d]

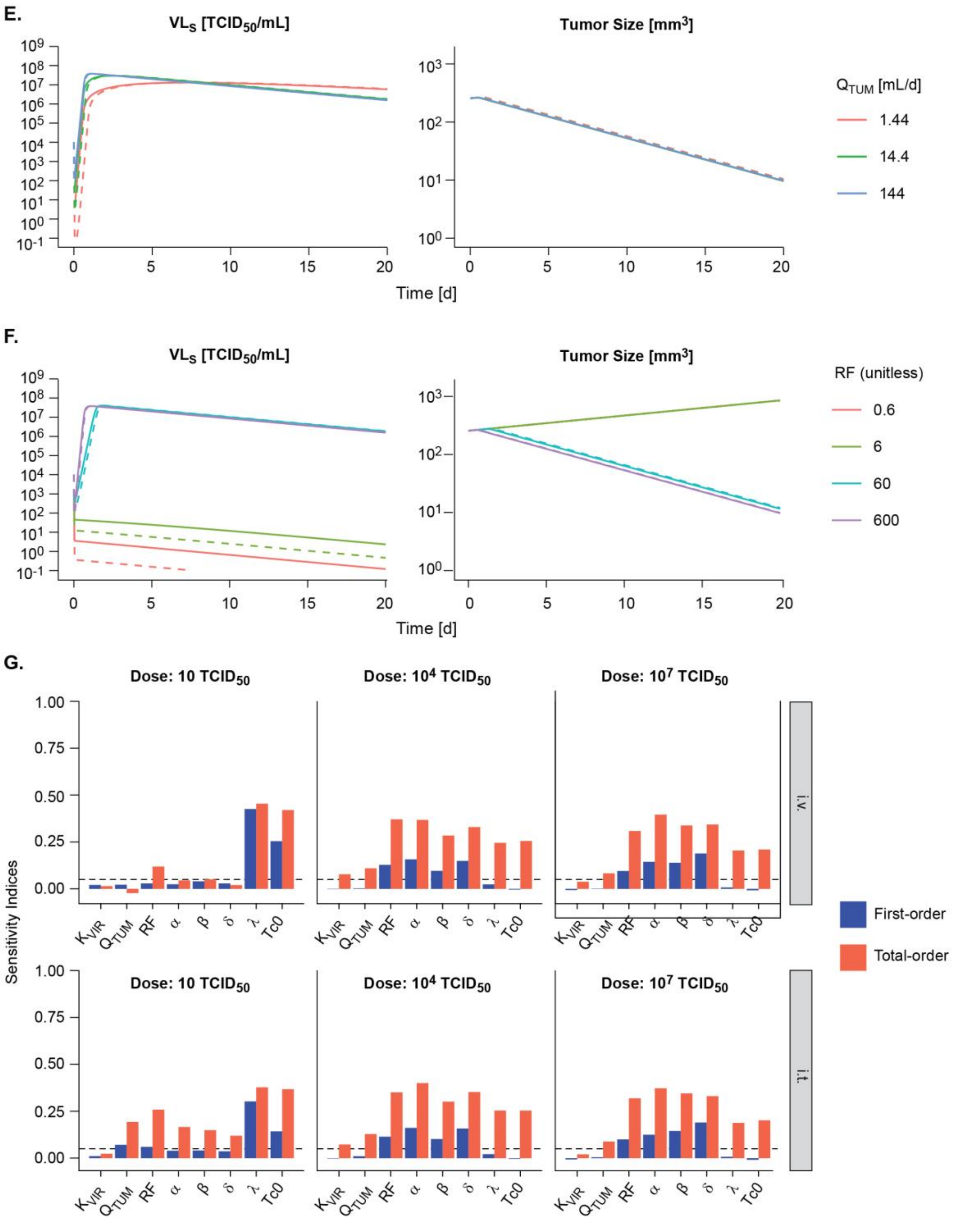

Supplementary Figure 4. Parameter Scan. Predicted time course of viral concentrations in serum (VL_S) and tumor size volume (lower panel) when A) viral infectivity (β), B) death of infected cells (δ), C) growth of tumor cells (λ), D) viral degradation in serum (K_{VIR}), E) tumor blood flow (Q_{TUM}) or F) viral tumor retention factor (RF) model parameters are varied one at a time over a plausible range of values after intravenous (dashed line) or intratumoral (solid line) administration of a dose of 10^4 $TCID_{50}$. G) First-order and total-order Sobol's sensitivity indices computed using model predicted tumor size at day 14 following intravenous (i.v.) or intratumoral
