# Supplementary material for: Mechanistic Modeling of a Novel Oncolytic Virus, V937, to Describe Viral Kinetic and Dynamic Processes Following Intratumoral and Intravenous Administration
Source: Front Pharmacol. 2021 Jul 23;12:705443. doi: 10.3389/fphar.2021.705443 (PMC8343024; doi:10.3389/fphar.2021.705443)
Supplement: Supplementary file 4 [file DataSheet1.PDF]

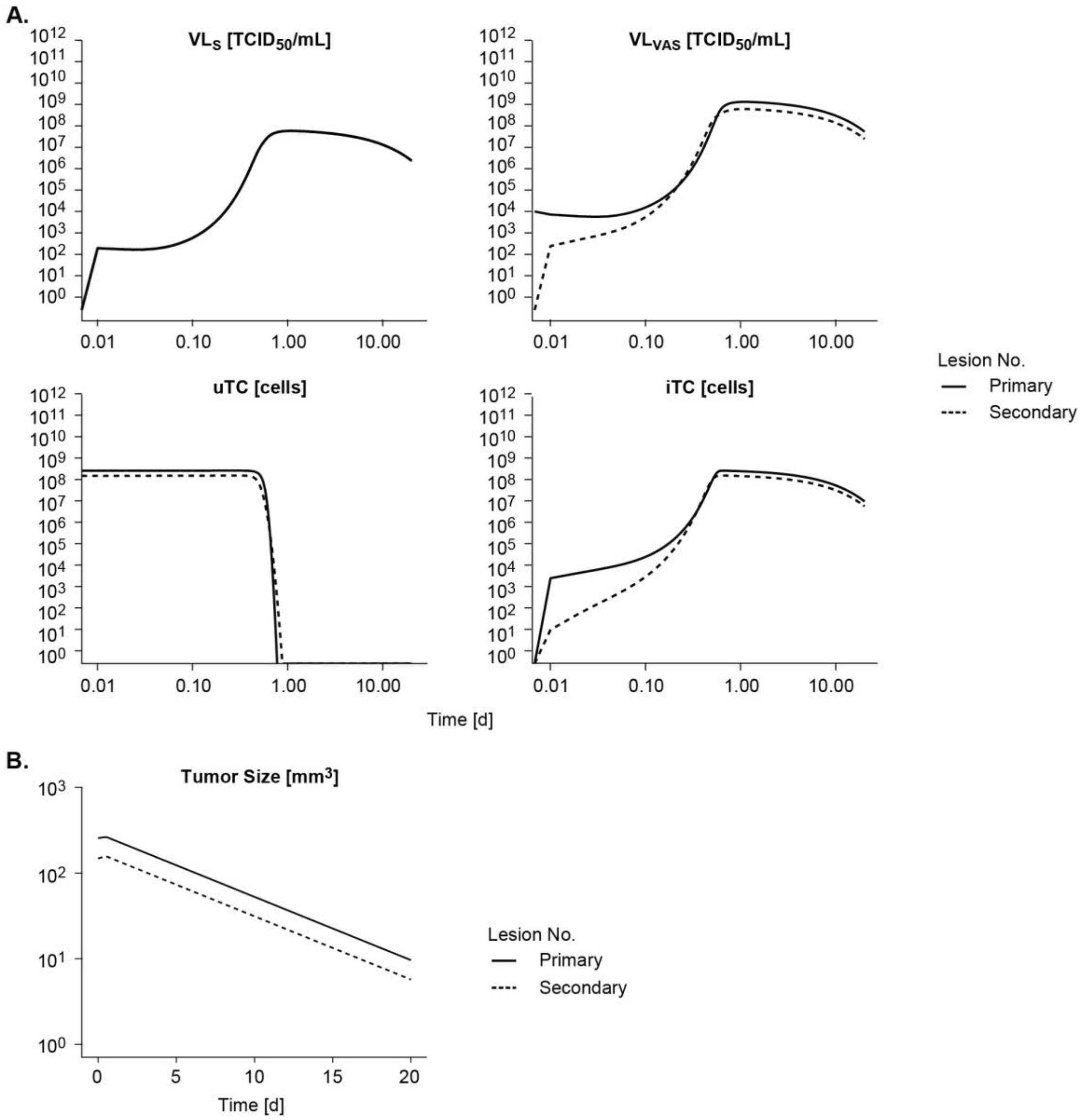

Supplementary Figure 2: Model predictions in mice bearing 2 tumor lesions. Predicted time course of (A) the different model entities or (B) the model predicted tumor size (mm^3) in the primary (solid line) and secondary (dashed line) lesion after administration of an intratumoral dose of 10^4 $TCID_{50}$ to the primary lesion. VL_S: viral load in serum, $VL_{INTRACELLULAR}$: viral load in tumor cells, uTC: uninfected tumor cells, iTC: infected tumor cells. Log- log scale used in panel A only.
